# Identification of SNP Markers and Candidate Genes Associated with Major Agronomic Traits in *Coffea arabica*

**DOI:** 10.3390/plants13131876

**Published:** 2024-07-07

**Authors:** Ruane Alice da Silva, Eveline Teixeira Caixeta, Letícia de Faria Silva, Tiago Vieira Sousa, Pedro Ricardo Rossi Marques Barreiros, Antonio Carlos Baião de Oliveira, Antonio Alves Pereira, Cynthia Aparecida Valiati Barreto, Moysés Nascimento

**Affiliations:** 1 Biotechnology Applied to Agriculture Institute (Bioagro), Federal University of Viçosa (UFV), Viçosa 36570-900, Brazil; ruane.alice29@gmail.com (R.A.d.S.); leticiafaria785@gmail.com (L.d.F.S.); pedro.barreirosufv@gmail.com (P.R.R.M.B.); 2 Embrapa Coffee, Brazilian Agricultural Research Corporation (Embrapa), Brasília 70770-901, Brazil; antonio.baiao@embrapa.br; 3 Biological Sciences Center, Iturama University Campus, Universidade Federal do Triângulo Mineiro (UFTM), Iturama 38025-180, Brazil; tiago.sousa@uftm.edu.br; 4 Agricultural Research Company of Minas Gerais (EPAMIG), Viçosa 36571-000, Brazil; tonico.epamig@gmail.com; 5 Laboratory of Intelligence Computational and Statistical Learning (LICAE), Department of Statistics, Federal University of Viçosa, Viçosa 36570-900, Brazil; cynthia.barreto@ufv.br (C.A.V.B.); moysesnascim@ufv.br (M.N.)

**Keywords:** coffee, genetic breeding, molecular markers, candidate genes, morpho agronomic traits

## Abstract

Genome-wide association studies (GWASs) allow for inferences about the relationships between genomic variants and phenotypic traits in natural or breeding populations. However, few have used this methodology in *Coffea arabica*. We aimed to identify chromosomal regions with significant associations between SNP markers and agronomic traits in *C. arabica*. We used a coffee panel consisting of 195 plants derived from 13 families in F2 generations and backcrosses of crosses between leaf rust-susceptible and -resistant genotypes. The plants were phenotyped for 18 agronomic markers and genotyped for 21,211 SNP markers. A GWAS enabled the identification of 110 SNPs with significant associations (*p* < 0.05) for several agronomic traits in *C. arabica*: plant height, plagiotropic branch length, number of vegetative nodes, canopy diameter, fruit size, cercosporiosis incidence, and rust incidence. The effects of each SNP marker associated with the traits were analyzed, such that they can be used for molecular marker-assisted selection. For the first time, a GWAS was used for these important agronomic traits in *C. arabica*, enabling applications in accelerated coffee breeding through marker-assisted selection and ensuring greater efficiency and time reduction. Furthermore, our findings provide preliminary knowledge to further confirm the genomic loci and potential candidate genes contributing to various structural and disease-related traits of *C. arabica*.

## 1. Introduction

Coffee is the second most consumed beverage worldwide, with Brazil at the top of the ranking as the world’s largest coffee producer and exporter [1]. The Brazilian scenario, with its variety of soil and climate conditions, provides a diversity of flavors for the beverage, making the product even more differentiated and able to meet the demand of domestic and foreign markets [2]. In the genus *Coffea*, *Coffea arabica* and *Coffea canephora* are the two most economically important species [3], both of which are cultivated in Brazil.

Given the importance of the coffee crop, innovative research is essential for enhancing profitability and sustainability to overcome the challenges of global coffee production. Genetic breeding plays a significant role in driving advancements in coffee cultivation through the creation cultivars that meet producer and market demands [4,5,6]. However, the conventional breeding process is time-consuming, multidisciplinary, and continuous. To make it faster and more efficient, the application of innovative tools is recommended, such as the use of biotechnology, which can contribute to the genetic progress of the crop [7,8,9].

*C. arabica* is allotetraploid [10] (2n = 4x = 44 chromosomes) and autogamous, and originated from the natural crossing of the diploid species *C. canephora* and *Coffea eugenioides* [11,12]. It is the most widely cultivated species of the *Coffea* genus. However, *C. arabica*, mainly due to its origin and high degrees of self-pollination and evolution, presents a narrow genetic base [13,14], making the breeding process difficult.

With its well-consolidated breeding programs, Brazil stands out for the number of coffee cultivars registered. However, according to Setotaw et al. (2013) [15], only seven ancestors contribute to more than 97% of the genetic base of Brazilian *C. arabica* cultivars, making it difficult to discriminate genotypes in breeding programs. This discrimination is especially limited due to the fact that quantitative traits, governed by many genes and greatly affected by the environment, are not classified into different phenotypic groups, which makes visual selection difficult [16].

Molecular markers have been used as an important tool to assist in breeding programs [7]. Among the types of molecular markers used in *C. arabica* for different purposes [2,17,18,19,20], SNP markers stand out for their ease of automation. This feature makes molecular markers suitable for large-scale genotyping, while the high-coverage and high-resolution markers can greatly improve the efficiency of breeding programs. Additionally, SNP markers offer a wealth of information due to their abundance in the genome [8,21].

The information obtained by means of SNP markers, together with phenotypic information, can be used in genome-wide association studies (GWASs) which, by means of hypothesis tests, allow for the detection of signification associations between the markers and complex traits [22]. These significant markers can be used to narrow down genomic regions and potential candidate genes within the SNP region that contribute to the traits of interest [23]. In addition, GWASs minimize the limitations previously encountered in traditional QTL mapping, as they allow for higher resolution and can be performed in natural populations which, for QTL mapping, is only possible in bi-parental crosses [24].

The efficiency of GWASs has been reported in several works, and they have been applied to different crops and traits of economic importance. In *C. arabica*, few studies have been conducted using the GWAS approach. These works include traits related to bean chemical composition for coffee beverage quality [25,26], the evolutionary history of MADS-box genes [27], and resistance to *Colletotrichum kahawae* [28]. Therefore, studies involving other traits of interest for coffee are important and necessary in order to understand and subsequently explore the low variability existing in this species, maximize genetic gains, and make coffee breeding programs faster and more efficient.

The objective of this study was to identify genomic regions and SNP markers associated with phenotypic traits of agronomic importance for *C. arabica* using a GWAS, which will assist in marker-assisted selection (MAS) for breeding programs to improve the desired traits.

## 2. Results

### 2.1. Phenotypic Data Evaluation

The means, standard deviations (STDs), and ranges of the evaluated traits are summarized in Table 1. The trait means ranged from 0.10 to 21.50 liters per plant for yield (Y); 2.00 to 17.90 cm for leaf length (LL); 2.40 to 7.90 cm for leaf width (LW); 26.20 to 120.00 cm for branch length (BL); 1 to 22 for the number of vegetative nodes (NVN); 1 to 18 for the number of reproductive nodes (NRN); 1 to 268 for the total number of fruits (NF); 0.2 to 675 for fruit volume (FV); 90.00 to 273.10 cm for plant height (PH); 1.32 to 9.54 cm for canopy diameter (CD); and 1.33 to 230.20 cm for stem diameter (SD). Relatively high heritability (h^2^) was estimated for most traits evaluated, ranging from 0.42 to 0.90, and PH and CD showed higher values. The heritability was moderate for MU (0.30), Cer (0.38), and LM (0.30), and the lowest for SD (0.01).

**Figure 1:**
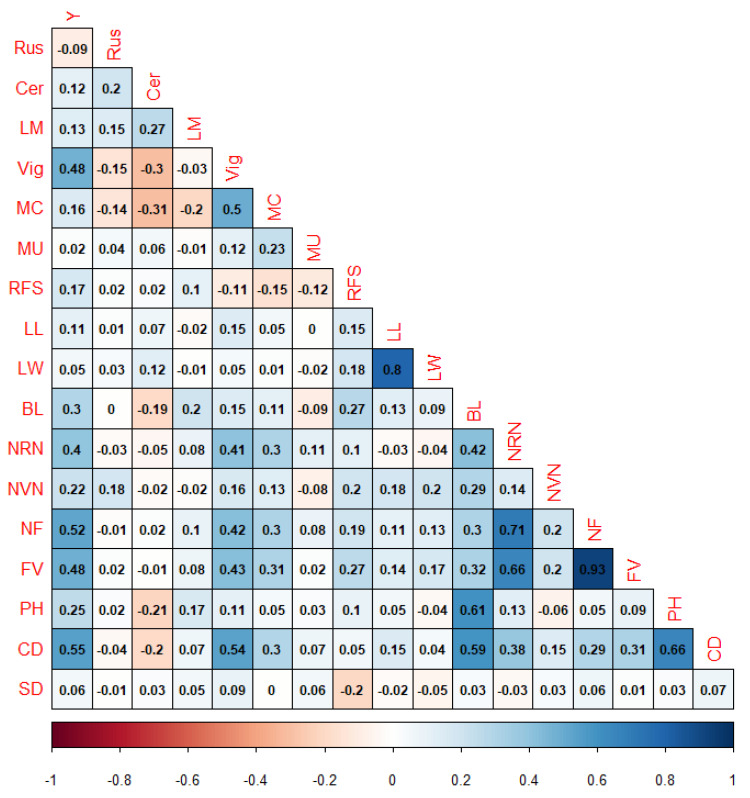
Pearson’s correlation between the adjusted phenotypic values. Y: yield; LL: leaf length; LW: leaf width; BL: branch length; NRN: number of reproductive nodes; NVN: number of vegetative nodes; NF: total number of fruits; FV: fruit volume; PH: plant height; CD: canopy diameter; SD: stem diameter; RFS: ripening fruit size; MU: maturation uniformity; MC: maturation cycle; Rus: rust incidence; Cer: cercosporiosis incidence; LM: leaf miner infestation; Vig: vegetative vigor.

The adjusted phenotypic values used for the GWAS presented normal distributions for all evaluated traits (Figures S1–S3). Overall, the Pearson’s correlations between the adjusted phenotypic values varied from low to high. The lowest Pearson’s correlation was estimated between the MC and Cer (0.31), while the highest Pearson’s correlations were estimated between the FV and NF (0.93) and the LL and LW (0.8) (Figure 1).

### 2.2. Structure Analysis

**Figure 2:**
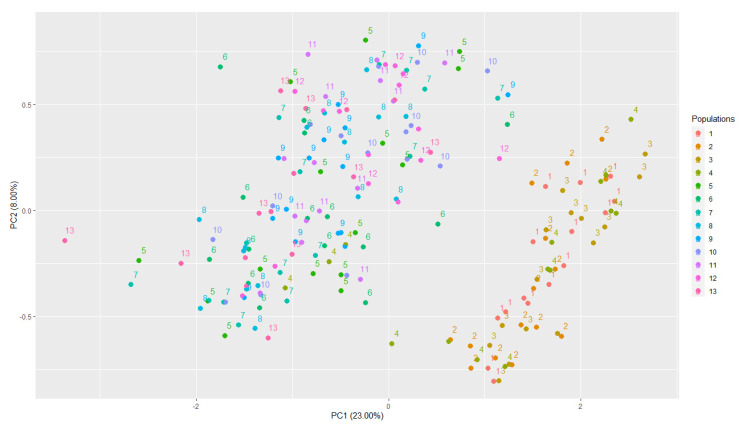
PCA of Coffea arabica panel: Scatter plot of the first two principal components (PC1 and PC2). Backcrossing (1 to 9) and selfing populations (10 to 13). (1) H 419-1 × UFV 445-46; (2) H 419-1 × Catuaí Amarelo IAC 30; (3) H 514-8 × UFV 440-10; (4) H 514-8 × Catuaí Amarelo IAC 86; (5) H 514-7 × UFV 440-10; (6) H 514-7 × Catuaí Amarelo IAC 86; (7) H 419-10 × UFV 445-46; (8) H 419-10 × Catuaí Amarelo IAC 30; (9) H 513-5 × Catuaí Amarelo IAC 64; (10) F_2_-H 419-10; (11) F_2_-H 514-8; (12) F_2_-H 514-7; (13) F_2_-H 513-5 (Table S1).

The genetic structure of the populations used in this study was assessed using principal component analysis (PCA) applied to the SNP dosage matrix (Figure 2). PC1 explained 23% of the variation in the genotypic data, whereas PC2 explained only 8% of the variation (Figure 2).

PCA analysis identified a diversity genetic difference among sample individuals (Figure 2). PCA was considered in GWAS analyses. The 13 progenies obtained from these crosses are shown in Table S1 and Figure S4.

### 2.3. SNP Marker Analysis

**Figure 3:**
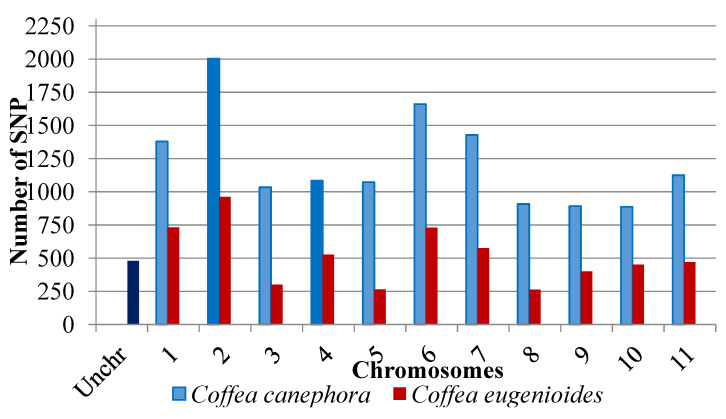
SNP molecular markers are distributed among the 11 chromosomes and the uncharacterized chromosome (Unchr) after alignment with the *C. arabica* genome. Blue bars correspond to SNPs mapped in the subgenome of *C. canephora*, and red bars in the *C. eugenioides* subgenome. These two subgenomes form the genome of the allotetraploid *C. arabica*.

After quality analysis, using the minor allele frequency (MAF) and the call rate as parameters, there was a reduction in the total number of SNPs markers, from 21,211 to 19,672. SNPs were located on all *C. arabica* chromosomes, in addition to an uncharacterized chromosome (UnChr). The number of SNPs per chromosome ranged from 479 (Unchr) to 2965 (chromosome 2) (Figure 3).

### 2.4. Genome-Wide Association Study

**Figure 4:**
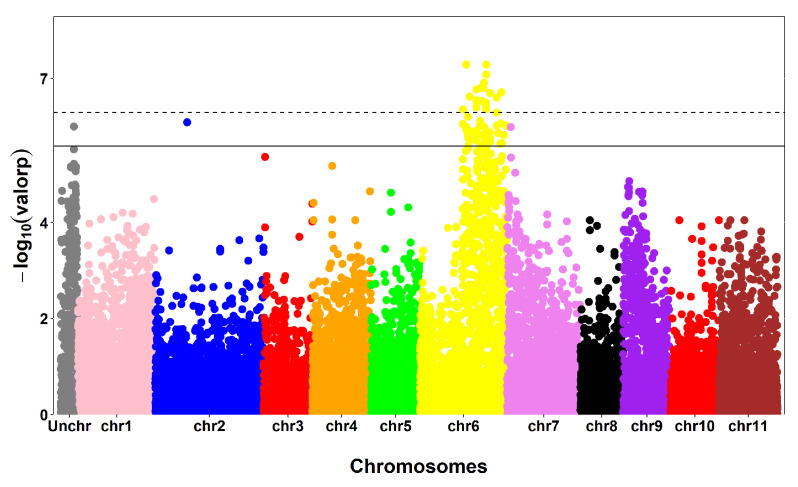
Manhattan plot for plant height. SNPs across the horizontal dashed line show significance with a moderately restricted threshold of −log10 (0.01/19,672). Graphs above the horizontal solid line show significance with a threshold of −log10 (0.05/19,672). The different colors indicate graphs for different chromosomes, which follow the following order: Unchr (uncharacterized chromosome) to chr11 (chromosome 11).

In total, 110 SNPs with significant associations at a 5% probability level and 42 SNPs with significant associations at 1% were detected for 7 of the 18 agronomic traits studied. The agronomic traits that showed significant associations were PH (56), BL (22), CD (12), NVN (3), RFS (2), Rus (1), and Cer (14). Among the 56 SNPs associated with the trait PH, 55 were found on chromosomes 2, 6, and 7 and an uncharacterized chromosome (Unchr) (Figure 4).

**Figure 5:**
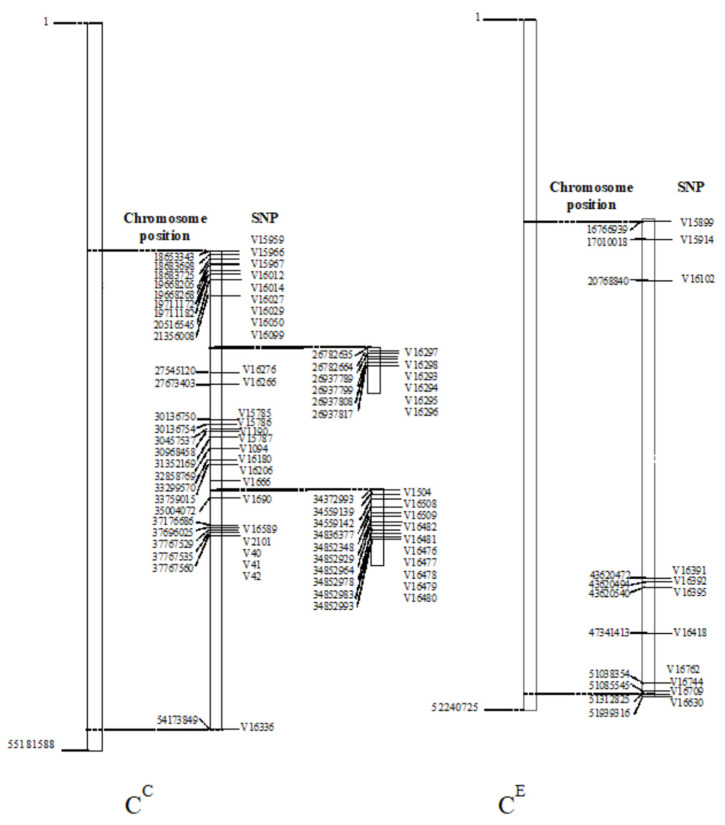
SNP positions on chromosome 6 of the C^C^ and C^E^ sub-genomes in the reference genome (GCA_003713225.1, NCBI). C^C^ represents the chromosome referring to the *C. canephora* subgenome, and C^E^ represents the part of the subgenome referring to *C. eugenioides*.

Chromosome 6 encompassed 96.36% of the total SNPs, and the remaining 3.64% were identified on chromosomes 2 and 7. Of the 53 SNPs located on chromosome 6, 42 SNPs were identified in the *C. canephora* subgenome, and 11 SNPs were identified in the *C. eugenioides* subgenome (Figure 5).

**Figure 6:**
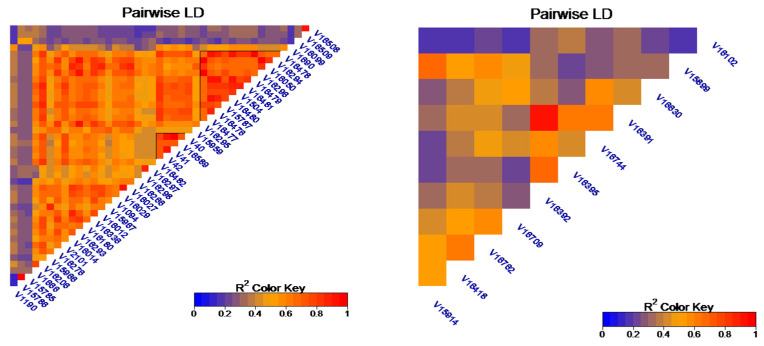
Linkage disequilibrium block formed by SNPs with significant associations for PH located in a region on chromosome 6 of *C. canephora* (**left**) and *C. eugenioides* (**right**). R^2^ values are indicated using the color intensity on the lower right side.

Given the high number of SNPs on chromosome 6, we performed linkage disequilibrium (LD) analysis with the significant SNPs for PH located on this chromosome. The analysis was performed for each chromosome set, one referring to *C. canephora*, and the other referring to *C. eugenioides* (Figure 6). In the chromosome set referring to the *C. eugenioides* subgenome, SNP V16391 and V16392 strongly stood out in the LD. In *C. arabica*, two LD blocks were identified on chromosome 6 of subgenome CC, with the larger block extending from SNP V16295 to V16478 and the smaller one extending from SNP C40 to V42 (Figure 6).

**Figure 7:**
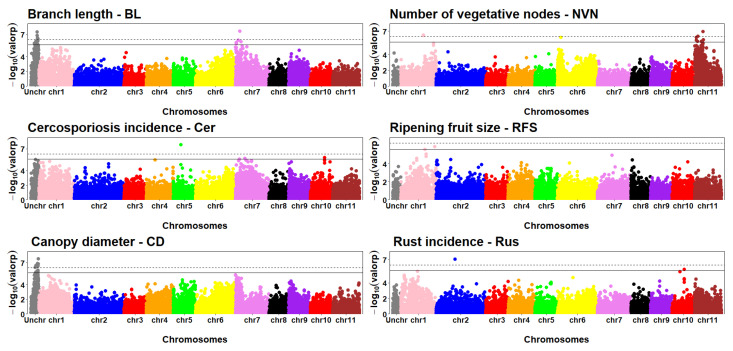
Manhattan plots for the traits. SNPs across the horizontal dashed line show significance with a moderately restricted threshold of −log_10_ (0.01/19,672). Graphs above the horizontal solid line show significance with a threshold of −log_10_ (0.05/19,672).

SNPs with significant associations were distributed on Chr7 and UnChr for BL; Chr1, Chr6 and Chr11 for NVN; Chr5, Chr7, and Chr10 for Cer; Chr2 and Chr10 for Rus; Chr1 for RFS; and UnChr for CD (Figure 7). Manhattan plots for the traits that did not present any significant SNPs are presented in Supplementary Figures S6 and S7. The QQ plot for assessing the GWAS qualities of traits with significant associations are presented in Figure S5.

### 2.5. Candidate Genes

The gene identification analysis based on National Center for Biotechnology Information (NCBI) data allowed for the detection of 16 SNPs associated with the PH corrected phenotype located in genes (Table S2). More than one SNP was detected in the same gene; for example, SNPs V16042, V15966, and V15967 were identified in a gene named LOC113691295. This gene is annotated as histidine—tRNA ligase, chloroplast/mitochondrial. SNPs V40, V41, and V42 were identified in gene LOC113693412, annotated as probable leucine-rich receptor-like protein kinase, at5g49770. SNPs V16508, V16509, V16630, and V17031 were found in genes with no functional annotation. The remaining SNPs were identified in genes LOC113692598, LOC113694028, LOC113697149, LOC113695058, LOC113695139, and LOC113696954. Their functional annotation is shown in Table S2.

For BL, the SNPs V17289 and V17241 were identified within genes; these SNPs were found in genes LOC113701091 and LOC113701577, respectively. According to NCBI, the gene LOC113701091 has no known function. The gene LOC113701577, located on chromosome 7 (C^E^), is described as a cold-regulated plasma membrane-type protein. For the increase in NVN, the SNPs that showed the highest percentage effects were the only ones identified in genes. The SNPs V4256 and V4257 were found in the same gene, LOC113713452, with no known function. SNP V14783 is in the LOC113697187 gene located on chromosome 6 (C^E^), annotated as an auxin transporter type 2 protein.

Three SNPs were associated with Cer. V5751 was on chromosome 10 (C^E^), located in the LOC113712673 gene, described as F-box protein/kelch repeat At1g57790; SNP V17709 was on chromosome 7 (C^E^), in the LOC113701115 gene, described as ubiquitin-conjugating enzyme E2 type 28 and SNP V13732 was located on chromosome 5 (C^E^), in the LOC113687823 gene, which corresponds to an MYB4 transcription factor.

Only SNP V2821 was identified as being in the genes for Rus. Located on chromosome 10 (C^E^), SNP V2821 was located in the LOC113711847 gene. On chromosome 10 of the subgenome EE, SNP V2821 was found in the uncharacterized gene (LOC113711847). For the CD trait, all SNPs with significant associations were located in the uncharacterized chromosome (Unchr).

## 3. Discussion

We performed a genome-wide association study for the main agronomic traits currently analyzed and considered in *C. arabica* breeding programs. A panel of 195 accessions from 13 coffee progenies were genotyped with 19,672 SNP markers and phenotyped for 18 traits.

Low to high heritabilities were estimated for the evaluated traits, ranging from 0.01 to 0.90. The lower value was observed for SD and the higher for PH and CD (Table 1). Similar results were observed by Rodrigues et al. (2017) [29], who found heritability values of 0.90 and 0.70 for PH and CD, respectively, in Arabica coffee genotypes. Getachew et al. (2017) [30] found lower heritability values than ours, of 0.28, 0.41, 0.47, and 0.52 for Y, LL, PH, and CD, respectively. The heritabilities of the LW and SD traits were higher, at 0.52 and 0.67, respectively.

The first two principal components, obtained using PCA, explained 31% of the total variance of the data. Variability was observed even in the narrow genetic base of coffee. The *C. arabica* genetic base is a consequence of a single polyploidization event in the origin of its tetraploid genome [14]. Moreover, in Brazil, only 13 ancestors are responsible for the genetic basis of the 121 coffee cultivars released between 1939 and 2009, evidencing the low genetic diversity among cultivars [15]. In our study, the populations analyzed using a GWAS have similar parents to these Brazilian cultivars, as they are considered the most important source for major agronomic traits in *C. arabica*. Even when using these cultivars as a parent, our populations showed enough variability to proceed with the GWAS.

In the genotypic data, we considered 19,672 SNP markers on different chromosomes and subgenomes. Even with the reduction, the number of SNPs used in our study was higher than that used in other *C. arabica* GWAS works. Sant’Ana et al. (2018) [25] used 6696 SNPs (2587 after filtering) to perform an association analysis for traits related to the chemical composition of the grain. Tran et al. (2018) [26] analyzed 1351 SNPs to identify markers associated with the caffeine and trigonelline biosynthesis pathways. Gimase et al. (2020) [28] examined 1635 SNPs and discovered two markers associated with resistance to *Colletotrichum kahawae*. The larger number of SNPs analyzed in the present study increases the possibility of identifying a greater number of regions important for the agronomic traits evaluated.

The combined analysis of genotyping and coffee reference genome allowed for the identification of SNPs widely distributed among all chromosomes. The SNP distribution among the chromosomes of *C. arabica* was inferred considering an allotetraploid nature of this species [10]. *C. arabica* is derived from the natural crossing between *C. canephora* and *C. eugenioides* [11]; accordingly, its genome is divided into two subgenomes, one referring to *C. canephora* and the other referring to *C. eugenioides* (Figure 3). In the coffee reference genome database, some scaffolds were not clustered on any of the chromosomes. These scaffolds were grouped into a set called uncharacterized chromosomes (Unchr).

Genome-wide association analysis identifies 110 significant SNPs (q-value < 0.05, Bonferroni correction) for the traits PH, BL, CD, NVN, RFS, Rus, and Cer. The GWAS captures a small proportion of genotypic variation as it evaluates each allele individually, so the methodology may be limited for some traits with more complex heritabilities [31]. In this case, the trait may be controlled by many rare alleles, each of which has a large effect on the phenotype, or it may have many common alleles with small effects, and these cannot be detected [32].

A total of 56 SNPs with significant associations were identified for the plant height (PH) trait. In general, most of the significant SNPs were located on chromosome 6, and the genomic information came from the two subgenomes of *C. arabica*. These results indicate that the two subgenomes control this trait. The large fraction of SNPs found on chromosome 6 suggests a probable QTL that controls this trait. Among the SNPs with significant associations for PH, 16 (14 on chromosome 6, 1 on chromosome 2, and another on chromosome 7) were found within the genes. However, SNPs not inserted within the genes (40 SNPs) are also relevant for use as genetic markers in breeding. A molecular marker does not necessarily need to be inserted in a gene to detect genetic differences among individuals; it can be associated with a gene and be efficient [33]. Furthermore, SNPs may be in promoter or regulatory regions and, therefore, involved in gene expression. From a phytotechnical point of view, PH is an important trait as shorter plants can be more efficient for harvesting or picking fruits [34].

Based on the functional annotation of the genes in which the SNPs were located, no known mechanism with direct influence on the control of plant height was identified. This can be explained by the complexity of the trait, indicating the presence of more genes acting on its control. According to Sousa et al. (2019) [8], who evaluated this trait in the same populations of *C. arabica*, plant height showed a moderate value of genomic heritability (0.46) and a high number of estimated QTLs (202). These values confirm the complexity of the trait. Moncada et al. (2016) [35] detected two QTLs associated with plant height in their work, located in linkage groups 1 and 4, but confirmed the high interference of the QTL × environment interaction component. This information corroborates the complexity of the genetic mechanisms involved in coffee height.

Among the identified SNPs, V1190 and V16099 had the greatest reduction effects on PH, reducing it by 21.21% and 19.56%, respectively. The reduction was evaluated as the ratio between the adjusted phenotypic means of individuals possessing one of the specific alleles and the entire population. With this approach, the percentage of the effect of the desired allele in the PH improvement was determined. In general, stronger associations are more useful in breeding programs [36].

The GWAS also detected 22 SNPs associated with the plagiotropic branch length (BL); however, 15 were not analyzed as they were located on scaffolds (*Unchr*) of the reference genome. The remaining five SNPs were located on chromosome 7, and the desirable allele was the one that increased the BL. For coffee trees, increasing the length of plagiotropic branches can result in increased productivity. Previous studies have demonstrated that the length of the plagiotropic branch is positively correlated with production [37,38]. The SNP V17241 was found in the LOC113701577 gene and is described as a cold-regulated plasma membrane-like protein. This gene is involved in the response of plants to low temperatures, mainly because low-temperature stress can influence the composition of the plasma membrane, reducing fluidity and providing increased rigidity of the cell membrane [39,40]. SNPs V17289 and V17241 showed an effect of increased BL when they were in heterozygosity. This result suggests a possible heterosis effect for these loci.

A total of 12 SNPs, distributed across three distinct chromosomes, were found to be associated with the number of vegetative nodes (NVNs). Of these, three SNPs were identified as being located within genes, which showed the highest percentage effects associated with the increase in the number of vegetative nodes. Only the gene containing the SNP V14783 has a known function: auxin transporter type protein 2. Auxin is a phytohormone that is directly related to the regulation of growth in plants. It is found in higher concentrations in the apex of plants, and may indirectly supply the development of lateral buds [41]. Thus, the concentration of this phytohormone is critical for the development of the number of vegetative nodes. Furthermore, like BL, the number of vegetative nodes also presents a positive correlation with production [4,38] and, therefore, is fundamental for coffee tree breeding programs.

Resistance to *Cercospora coffeicola*, the etiological agent of brown eye spot disease in coffee, has been sought in genetic breeding programs for this crop since its incidence has increased with climate change. As a consequence, the losses in recent years have had a great impact on the production of seedlings in nurseries [33]. All SNPs associated with a reduced incidence of *C. coffeicola* are located within genes. SNP V5751 was associated with the gene described as F-box protein/kelch repeat At1g57790, and SNP V17709 with the gene described as ubiquitin-conjugating enzyme E2 type 28. According to Akter et al. (2014) [42], these genes are involved in antibacterial defense mechanisms; however, other genes may be associated with factors that reduce the incidence of fungal diseases, such as cercosporiosis. However, further studies are needed to confirm the function and mode of action of these genes in the control of *C. coffeicola* in coffee trees. SNP V13732 was inserted in a gene corresponding to a MYB4 transcription factor. Transcription factors act as regulators of gene expression, leading to gene activation. Furthermore, the MYB transcription factor family is involved in the regulation of plant tolerance to biotic and abiotic stresses [43]. Therefore, this is an SNP associated with a reduced incidence of *C. coffeicola*, which should be validated and may result in a functional marker, contributing to the advancement of breeding programs for this species. This is especially true given the lack of validated molecular markers linked to resistance to this disease in the literature.

Two SNPs had a significant association with rust incidence (Figure 7). Rust caused by the biotrophic fungus *Hemileia vastatrix* is the most economically important disease affecting coffee crops worldwide [44]. Thus, the search for sources of resistance to this disease is one of the main objectives of coffee breeding programs. Only SNP V2821 was found to be inserted in a gene. However, there was no characterized functional annotation for this gene.

One SNP, V4722, was significantly associated with the fruit size, showing an increase of 16.93%. Larger coffee fruits are connected with bean quality. Among the factors that affect coffee quality are the physical aspects of the beans, including fruit size, which affects the price of the product and ensures higher revenues to growers. Moreover, the size of the beans is one of the factors that determine the productive potential of cultivars, and their homogeneity ensures a more uniform roasting [45]. For the phenotypic trait canopy diameter, 14 SNPs were obtained with significant association; however, none of them were recognized within genes.

## 4. Materials and Methods

### 4.1. Plant Materials and Experimental Conditions

The population used for genotyping and phenotyping corresponds to 13 progenies originated from crosses between resistant and susceptible coffees to leaf rust (*Hemileia vastatrix*). Three parents belonging to the Catuaí cultivars group (Catuaí Amarelo IAC 30, IAC 86, and IAC 64, susceptible to *H. vastatrix*) and three parents from the Híbrido de Timor group (HdT) (UFV 445-46, UFV 440-10, and UFV 530, resistant to *H. vastatrix*) were crossed. Five F_1_ hybrids, H 419-1, H 419-10, H 513-5, H 514-7, and H 514-8, were selected based on rust resistance and other desirable agronomic traits. These F_1_ were selfed and backcrossed, resulting in a population with resistant backcrosses (BCr), susceptible backcrosses (BCs), and F_2_ generations. In each progeny, 15 genotypes were selected, totaling 195 individuals (Table S1 and Figure S4).

The field experiment was installed on February 11, 2011, at the experimental station of the Department of Plant Pathology, Universidade Federal de Viçosa, Brazil (20°44′25″ S; 42°50′52″ W). The plants were arranged at a spacing of 3.0 m between rows and 0.7 between plants. Federer’s augmented blocks [46] were used as a statistical design, consisting of three blocks with 65 regular treatments and two controls (Catuaí Vermelho IAC 44 and Oeiras MG 6851). Two plants per controls were randomly assigned within each block. Liming and fertilization were performed according to crop requirements, and no phytosanitary control methods were used to control rust (*H. vastatrix*), anthracnose (*Colletotrichum kahawae*), or the leaf miner bug (*Leucoptera coffeella*).

### 4.2. Phenotypic Evaluation

The phenotypic evaluations were performed in 2014, 2015, and 2016. In each year, 18 phenotypic traits of agronomic importance were evaluated. Among the traits, 11 were continuous and evaluated from measurements: yield (Y), leaf length (LL), leaf width (LW), branch length (BL), number of reproductive nodes (NRN), number of vegetative nodes (NVN), total number of fruits (NF), fruit volume (FV), plant height (PH), canopy diameter (CD), and stem diameter (SD). The other traits—ripening fruit size (RFS), maturation uniformity (MU), maturation cycle (MC), rust incidence (Rus), cercosporiosis incidence (Cer), leaf miner infestation (LM), and vegetative vigor (Vig)—were evaluated categorically, using a scale of grades. Descriptions of how the evaluations of each trait were performed are shown in Sousa et al. (2019) [8] and Table 2.

Phenotypic data were corrected for effects of years, plots, and year x plot interaction. The analyses were performed considering linear mixed models (REML/BLUP procedure), using the software Selegen-REML/BLUP [47] (https://ppestbio.ufv.br/marcos-deon-vilela-de-resende/) Accessed in 30 May 2024. Genetic parameters were estimated from the individual analysis of the 18 traits using the following statistical model:

y=Xu+Zg+Wp+Vr+Tb+Ri+e

where:

*y*: Equals the data vector;
*u*: Equals the vector referring to the overall average in each evaluation year;
*g*: Equals the vector of progeny effects (random effect—$g\;\sim N\left(0,\sigma_{g}^{2}\right))$;
*p*: Equals the permanent variance between plants (random effect—$p\;\sim N\left(0,\sigma_{p}^{2}\right)$);
*r*: Equals the variance between types of populations (random effect—$r\;\sim N\left(0,\sigma_{r}^{2}\right)$);
*b*: Equals the between-plot variance (random effect—$b\;\sim N\left(0,\sigma_{b}^{2}\right)$);
*i*: Equals the variance of the interaction progenies × years (random effect—$i\;\sim N\left(0,\sigma_{i}^{2}\right)$);
*e*: Equals the vector of residuals (random effect—$e\;\sim N\left(0,\sigma_{e}^{2}\right)$).

The capital letters represent the incidence matrices for the mentioned effects.

The individual heritability was defined by: $h^{2}=\sigma_{g}^{2}/\left(\sigma_{g}^{2}+\sigma_{p}^{2}+\sigma_{r}^{2}+\sigma_{b}^{2}+\sigma_{i}^{2}+\sigma_{e}^{2}\right)$.

### 4.3. DNA Extraction and Genotyping

The extraction of genomic DNA was performed according to Sousa et al. (2019) [8]. DNA was extracted from young and fully expanded leaves of the 195 *C. arabica* genotypes using the methodology proposed by Diniz et al. (2005) [48], in the Laboratory of Coffee Biotechnology—Biocafé/UFV. The DNA concentration was determined using NanoDrop2000, and the quality was determined using 1% agarose gel. After the standardization of the DNA concentration of the samples, they were sent to RAPiD Genomics, located in Florida, USA, for the construction of probes, sequencing, and identification of SNP molecular markers. The strategy used was based on the target genome capture of regions in the coffee genome, followed by next-generation sequencing. Sequencing data were cleaned and trimmed using Trimmomatic. The SNP identification was performed with the RAPiD Genomics, using the Capture-Seq pipeline and the Caturra genome available in the NCBI (RefSeq assembly GCF_003713225.1).

### 4.4. Quality Control of Molecular Markers

A total of 40,000 DNA probes were constructed for the SNP marker identification and genotyping of coffee trees. These regions were bioinformatically identified to avoid repetitive elements and to screen a large number of annotated genes. To capture these regions, we designed probes using a combination of genomic resources, including the *C. canephora* reference genome (https://coffee-genome-hub.southgreen.fr/, accessed on 4 July 2021), and assembled unigenes specific to each of the two species [49]. Of these, 10,000 polymorphic probes were selected. Further information about the construction of the probes and the identification of SNPs can be found in Sousa et al. (2017) [18].

The 195 coffee trees were genotyped using the 10,000 probes and the *Capture-Seq* methodology (*RAPiD genomics*), resulting in 19,672 SNP markers after quality analysis. The quality parameters adopted were a CR (call rate) equal to or greater than 90% and a MAF (minor allele frequency) of 5%. For the MAF parameter, the critical level was established using the equation $MAF=\frac{1}{\sqrt{2N}}$, where N refers to the number of individuals evaluated. Another quality criterion performed was the elimination of false SNPs, which can occur due to the polyploidy of *C. arabica* species. The strategy was to exclude SNPs that showed no variance among the individuals comprising the population under study, i.e., the heterozygosity was equal to 1.

To locate and verify the distribution of SNP markers in the *C. arabica* genome, a *BLAST* (Basic Local Alignment Search Tool) similarity analysis was performed between the sequences of the probes developed and the *C. arabica* genome (GCA_003713225.1) available at the NCBI (National Center for Biotechnology Information). Through the location of the sequence of each probe, the position of the SNP in the genomes of *C. arabica* was identified.

### 4.5. Genome-Wide Association Study (GWAS)

Genome-wide association analyses were performed using the SNP dataset, consisting of 19,672 SNPs. To perform the GWAS analyses, the genetic structure of the population was obtained with principal component analysis (PCA) using the *princomp* function of R software (3.4.3) [50]. The PCA methodology was applied to the genotypic data to obtain the principal components (PCs) that capture most of the genetic variation. This multivariate technique allows for the reduction of data mass with the least possible loss of information [51]. The principal components obtained were used as covariates in the GWAS model to detect SNPs associated with phenotypic traits. We retained the components that explained at least 70% of the total variation in the data.

The general GWAS model is given by:

$${Y_{ijk}}^{*}=\mu +{SNP}_{i}+\sum_{k=1}^{1}{PC}_{jk}+\varepsilon_{ijk}$$

where

${Y_{ijk}}^{*}$ is the adjusted phenotype;
μ is the population mean;
SNP*_i_* is the fixed effect of the *i*th SNP, adjusted as a covariate (allele substitution effect);
${PC}_{kj}$ is the fixed effect of the *k*th individual principal component K;
$\varepsilon_{ijk}$ is the error associated with ${Y_{ijk}}^{*}$.

The vector $\theta ={\left[\mu ,{SNP}_{i},{PC}_{jk}\right]}^{T}$ represents unknown parameters. The adopted thresholds were –log_10_(2.54 × 10^−6^) and –log_10_(5.08 × 10^−7^), calculated based on Bonferroni’s method correction for multiple testing (α∗α/m, where *m* is the total number of SNPs used in the association analysis), in order to identify SNPs significant at the 5% and 1% levels, respectively.

To quantify the effects of favorable alleles, according to the goal of the coffee program, the ratio between the phenotypic mean of individuals possessing a specific allele pair and the whole population was estimated:

Rz = (µ_s_/µ_g_) − 1 × 100

where

Rz is the ratio between the phenotypic mean of individuals possessing a specific pair of alleles and the whole population;
µ_s_ is the phenotypic mean of individuals selected for 0, 1, or 2 alleles;
µ_g_ is the general average of all individuals.

Linkage disequilibrium (LD) was estimated using the r^2^ parameter among SNPs located in the same region of the genome. LD analyses were performed using custom scripts in R software. The codes are available at https://github.com/licaeufv/GWAS-agronomic-traits-in-Coffea-arabica, accessed on 2 August 2021.

### 4.6. Putative Candidate Genes Analyses

The SNPs with significant associations identified in the GWAS were analyzed using custom scripts in Phyton v3.6 software for the identification of SNPs inserted within genes. Subsequently, the functional annotations of the genes containing the SNPs were analyzed using the *C. arabica* genome as a base.

## 5. Conclusions

The GWAS applied in our work allowed us to identify SNPs with significant associations in 7 out of 18 desirable agronomic traits of coffee. These SNP markers enable applications in accelerated coffee breeding through marker-assisted selection, ensuring greater efficiency and time reduction. The benefits of GWAS are maximized by the fact that *C. arabica* is a perennial crop and, therefore, has a high cost of maintaining populations in the field for phenotyping. Furthermore, our findings will help to provide advanced knowledge on the biological processes associated with important traits, as candidate genes were predicted with the associated SNPs.

## Figures and Tables

**Table 1:** Statistical summary of the evaluated traits of *C. arabica*.

Trait	h^2^	Minimum	Maximum	Mean	SD
Y	0.55	0.10	14.00	5.00	3.84
LL	0.42	2.00	17.90	12.16	1.79
LW	0.44	2.40	7.90	5.13	0.89
BL	0.78	30.80	120.00	66.42	15.02
NRN	0.49	1.00	185.00	8.52	8.24
NVN	0.44	1.00	22.00	9.31	2.68
NF	0.49	1.00	286.00	61.38	50.26
FV	0.57	0.40	225.00	119.13	72.21
PH	0.90	1.83	283.10	164.11	31.92
CD	0.90	1.33	229.10	148.57	29.26
SD	0.01	2.24	8.70	5.32	2.68
RFS	0.50	1.00	3.00	2.33	0.60
MU	0.30	1.00	4.00	2.71	0.78
MC	0.72	1.00	5.00	2.80	0.91
Rus	0.61	1.00	5.00	2.34	0.99
Cer	0.38	1.00	5.00	2.51	0.72
LM	0.30	1.00	4.00	2.06	0.69
Vig	0.70	4.00	10.00	7.23	1.19

Y: Yield; LL: Leaf length; LW: Leaf width; BL: Branch length; NRN: Number of reproductive nodes; NVN: Number of vegetative nodes; NF: Total number of fruits; FV: Fruit volume; PH: Plant height; CD: Canopy diameter; SD: Stem diameter; RFS: Ripening fruit size; MU: Maturation uniformity; MC: Maturation cycle; Rus: Rust incidence; Cer: Cercosporiosis incidence; LM: Leaf miner infestation; Vig: Vegetative vigor.

**Table 2:** Phenotypic traits evaluated in 2014, 2015, and 2016 in Viçosa (MG).

Traits		Type of Evaluation
Yield	(Y)	Liters of fresh cherries harvested per plant
Leaf length (cm)	(LL)	Measured in the leaf of the third or fourth pair of a plagiotropic branch of the middle third of the plant (cm)
Leaf width (cm)	(LW)	
Branch length (cm)	(BL)	Measured in the plagiotropic branch of the middle third of the plant
Number of reproductive nodes	(NRN)	
Number of vegetative nodes	(NVN)	
Total number of fruits	(NF)	
Fruit volume	(FV)	
Plant height (cm)	(PH)	Measured in the orthotropic branch (from the soil surface to the final branch growth point)
Canopy diameter (cm)	(CD)	Measured transversely to the planting row, considering the greatest canopy longest
Stem diameter (cm)	(SD)	Measured at the stem region of the plant (about 5 cm from the soil surface)
Ripening fruit size	(RFS)	Evaluated by a score scale ranging from 1 to 3
Maturation uniformity	(MU)	Evaluated by a score scale ranging from 1 to 4
Maturation cycle	(MC)	Evaluated by a score scale ranging from 1 to 5
Rust incidence	(Rus)	
Cercosporiosis incidence	(Cer)	
Leaf miner infestation	(LM)	
Vegetative vigor	(Vig)	Evaluated by a score scale ranging from 1 to 10

## Data Availability

The phenotypic data are originally from a study of Sousa et al. (2019) [8]. The SNP dataset used and identified can be accessed by the chromosome position (Table S2) deposited in the National Center of Biotechnology Information—NCBI (www.ncbi.nlm.nih.gov), BioProjectPRJNA506972.

## Supplementary Materials

The following supporting information can be downloaded at: https://www.mdpi.com/article/10.3390/plants13131876/s1, Table S1: *Coffea Arabica* progenies evaluated in 2014, 2015, and 2016 in Viçosa (Brazil). Table S2: Functional annotation of SNP inserts in genes for PH. Figure S1: Distribution of the adjusted phenotypic values used for the GWAS. Y: yield; LL: leaf length; LW: leaf width; BL: branch length; NRN: number of reproductive nodes; NVN: number of vegetative nodes. Figure S2: Distribution of the adjusted phenotypic values used for the GWAS. NF: total number of fruits; FV: fruit volume; PH: plant height; CD: canopy diameter; SD: stem diameter; RFS: ripening fruit size. Figure S3: Distribution of the adjusted phenotypic values used for the GWAS. MU: maturation uniformity; MC: maturation cycle; Rus: rust incidence; Cer: cercosporiosis incidence; LM: leaf miner infestation; Vig: vegetative vigor. Figure S4: Heredogram of the 13 progenies of Coffea arabica from crosses between parents of the Catuaí group and Híbrido de Timor (HdT); C1, C2, and C3 correspond to cultivars Catuaí Amarelo IAC 30, IAC 86, and IAC 64, respectively; HdT1, HdT2, and HdT3 correspond to the genotypes UFV 445-46, UFV 440-10, and UFV 530, respectively; H1, H2, H3, H4, and H5 are hybrids from crosses between the parents Catuaí Amarelo and HdT; 1, 3, 5, and 7 are progenies of the first rust-resistant backcross generation; 2, 4, 6, 8, and 9 are progenies of the first rust-resistant backcross generation; 10, 11, 12, and 13, progenies in the F2 generation. Figure S5: QQ plot for assessing the GWAS results of traits with significant associations. Figure S6: Manhattan plots for the traits. Y: yield; LM: leaf miner infestation; Vig: vegetative vigor; MC: maturation cycle; MU: maturation uniformity; LL: leaf length. Figure S7: Manhattan plots for the traits. LW: leaf width; NF: total number of fruits; FV: fruit volume; NRN: number of reproductive nodes; SD: stem diameter.
