# Supplementary material for: Identification of SNP Markers and Candidate Genes Associated with Major Agronomic Traits in Coffea arabica
Source: Plants (Basel). 2024 Jul 7;13(13):1876. doi: 10.3390/plants13131876 (PMC11243787; doi:10.3390/plants13131876)
Supplement: Supplementary file 1 [file plants-13-01876-s001.zip › plants-3059872-supplementary.pdf]

Table S1. *Coffea Arabica* progenies evaluated in 2014, 2015 and 2016 in Viçosa (Brazil)

Progenies	Progenitor 1	Progenitor 2
BCr 1	H 419-1 c-17	UFV 445-46
BCs 2	H 419-1 c-17	UFV 2143-235
RCr 3	H 514-8 c-387	UFV 440-10
BCs 4	H 514-8 c-387	UFV 2154-344
BCr 5	H 514-7 c-364	UFV 440-10
BCs 6	H 514-7 c-364	UFV 2154-344
BCr 7	H 419-10 c-214	UFV 445-46
BCs 8	H 419-10 c-214	UFV 2143-235
BCs 9	UFV 2148-57	H 513-5 c-14
F_2 10	H 514-8 c-387	-
F_2 11	H 514-7 c-364	-
F_2 12	H 419-10 c-214	-
F_2 13	H 513-5 c-14	-

BCr: First resistant backcross; BCs: First susceptible backcross; F_2: Generation obtained by the selfing of F_1 hybrids.

Table S2. Functional annotation of SNP inserts in genes for PH.

SNP	Chr	Position	Gene	Functional Annotation
V16042	chr2_e	12528092	LOC113691295	Histidine - tRNA ligase, chloroplast / mitochondrial
V15966	chr6_c	18683698	LOC113691295	Histidine - tRNA ligase, chloroplast / mitochondrial
V15967	chr6_c	18683725	LOC113691295	Histidine - tRNA ligase, chloroplast / mitochondrial
V16050	chr6_c	20516595	LOC113692598	IscA-like iron-sulfur assembly protein 2, mitochondrial
V16206	chr6_c	33299570	LOC113694028	Dihydroneopterin aldolase 1
V16508	chr6_c	34559139	LOC113693325	Uncharacterized
V16509	chr6_c	34559142	LOC113693325	Uncharacterized
V40	chr6_c	37767529	LOC113693412	Likely leucine-rich receptor-like protein kinase, at5g49770
V41	chr6_c	37767535	LOC113693412	Likely leucine-rich receptor-like protein kinase, at5g49770
V42	chr6_c	37767560	LOC113693412	Likely leucine-rich receptor-like protein kinase, at5g49770
V15899	chr6_e	16766939	LOC113697149	Strictosidine synthase 10-like protein
V15914	chr6_e	17010018	LOC113695058	Benzyl alcohol O-benzoyltransferase
V16744	chr6_e	51085545	LOC113695139	Putative serine/threonine/threonine protein type protein kinase CCR3
V16709	chr6_e	51312825	LOC113696954	B3 domain-containing protein, similar to At3g25182
V16630	chr6_e	51939316	LOC113697088	Uncharacterized
V17031	chr7_c	677592	LOC113699150	Uncharacterized

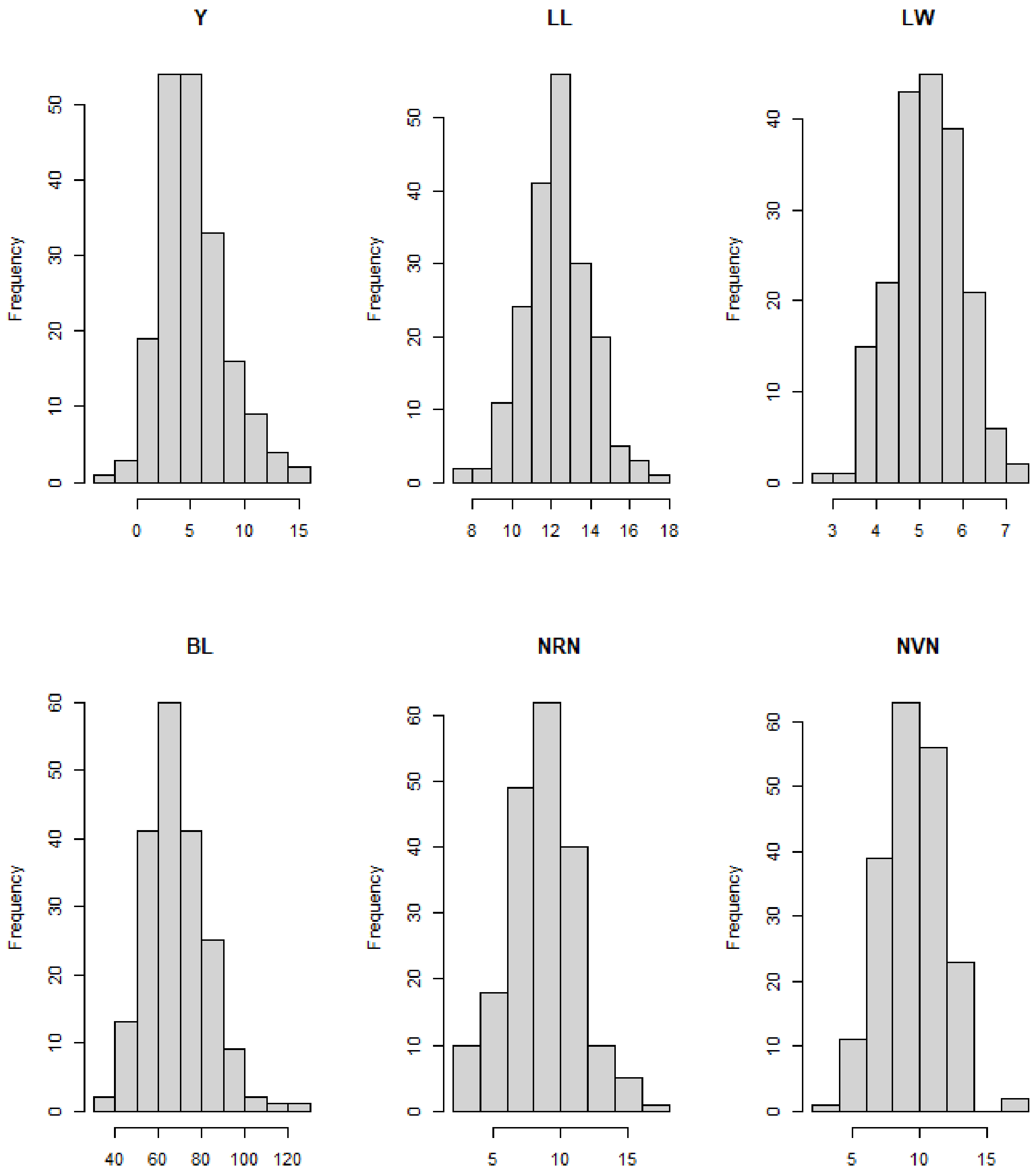

Figure S1. Distribution of the adjusted phenotypic values, used for the GWAS. Y: yield; LL: leaf length; LW: leaf width; BL: branch length; NRN: number of reproductive nodes; NVN: number of vegetative nodes.

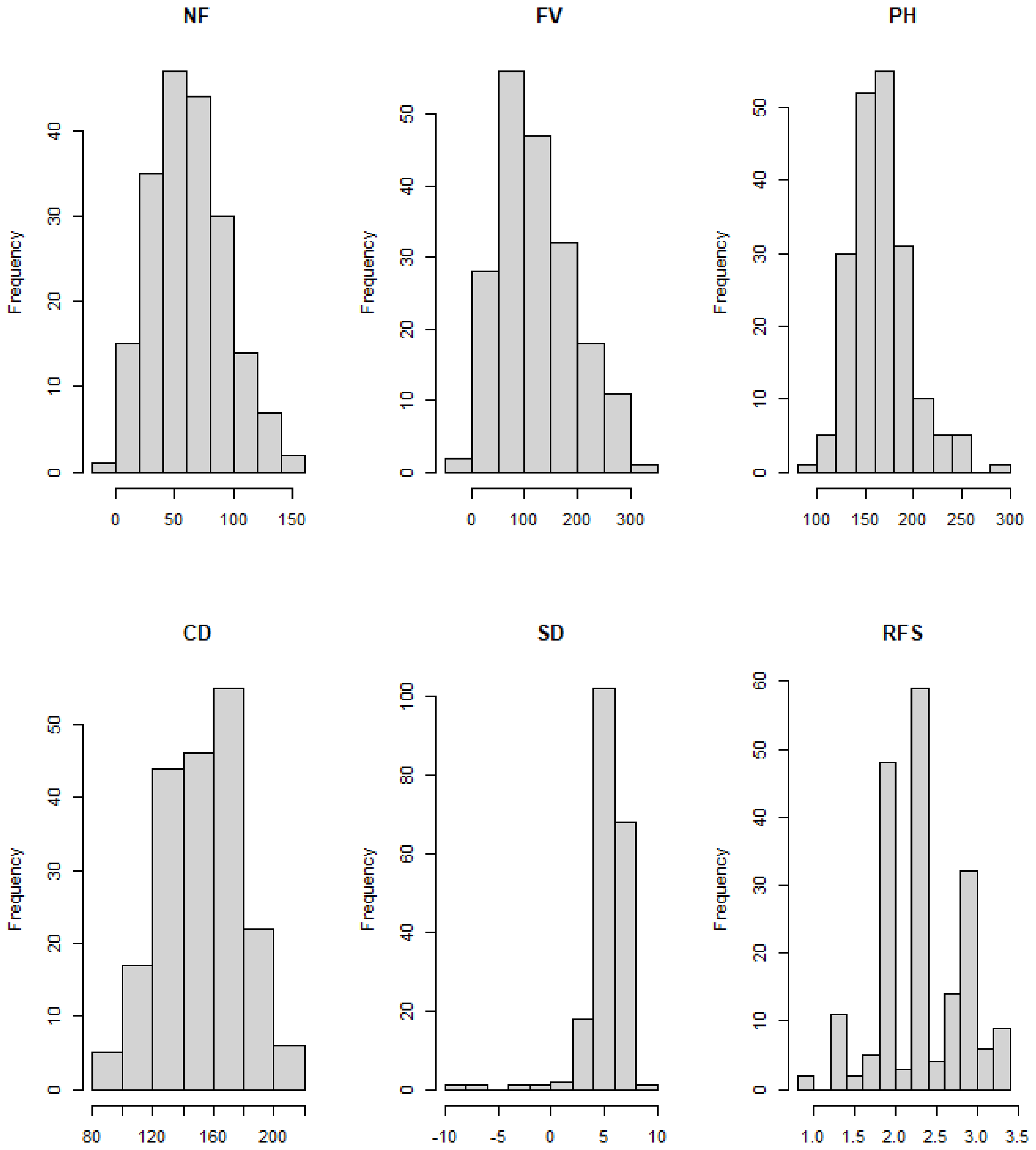

Figure S2. Distribution of the adjusted phenotypic values, used for the GWAS. NF: total number of fruits; FV: fruit volume; PH: plant height; CD: canopy diameter; SD: stem diameter; RFS: ripening fruit size.

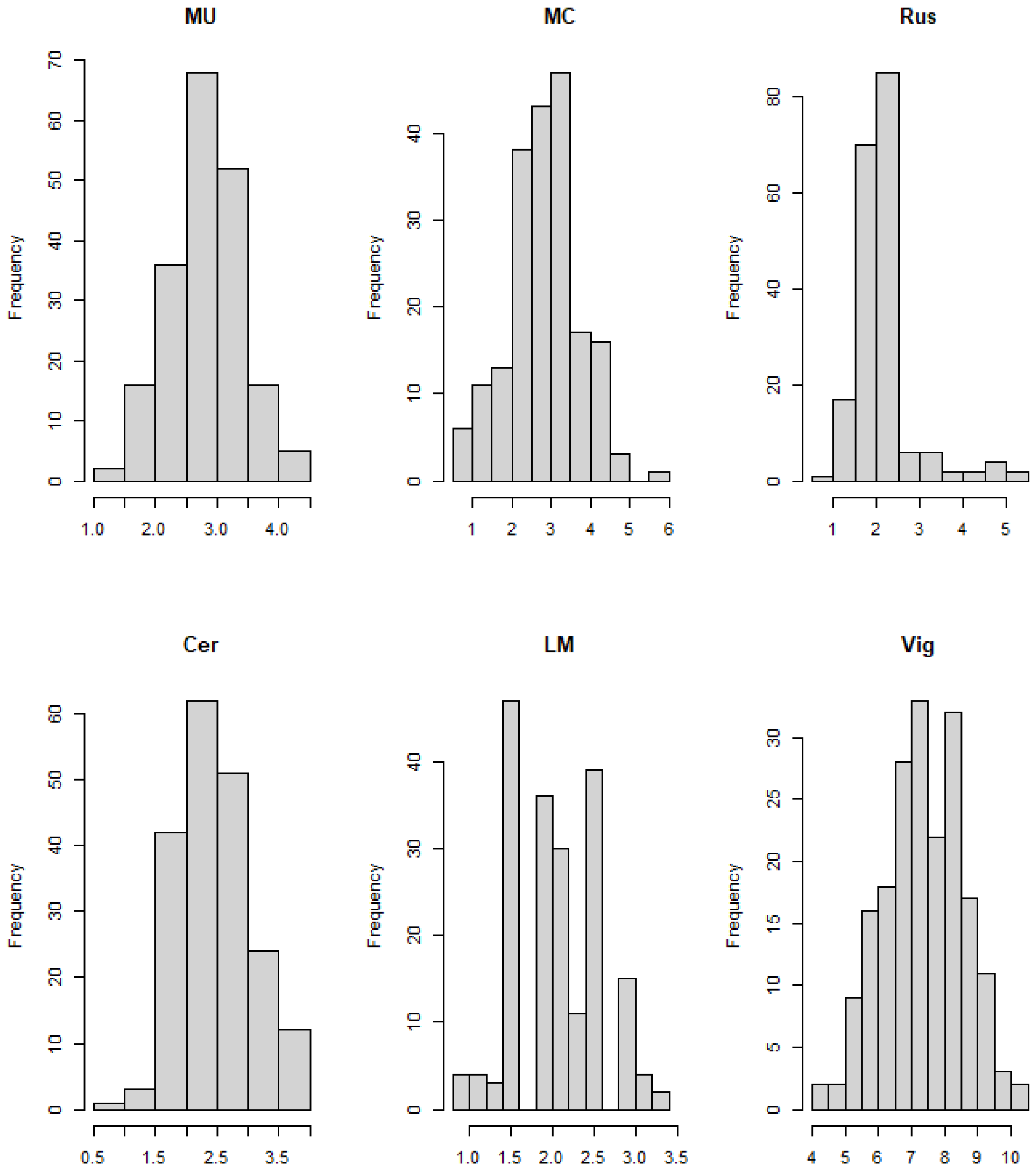

Figure S3. Distribution of the adjusted phenotypic values, used for the GWAS. MU: maturation uniformity; MC: maturation cycle; Rus: rust incidence; Cer: cercosporiosis incidence; LM: leaf miner infestation; Vig: vegetative vigor.

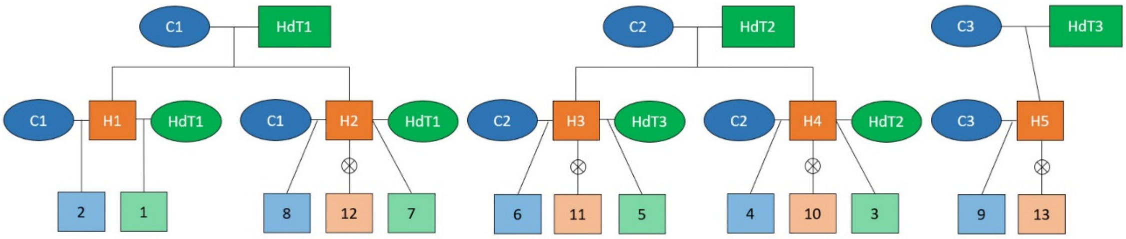

Figure S4. Heredogram of the 13 progenies of C*offea arabica* from crosses between parents of the Catuaí group and Híbrido de Timor (HdT); C1, C2, and C3 correspond to cultivars Catuaí Amarelo IAC 30, IAC 86, and IAC 64, respectively; HdT1, HdT2, and HdT3 corresponds to the genotypes UFV 445-46, UFV 440-10, and UFV 530, respectively; H1, H2, H3, H4, and H5 are hybrids from crosses between the parents Catuaí Amarelo and HdT; 1, 3, 5, and 7, are progenies of first rust-resistant backcross generation; 2, 4, 6, 8, and 9, progenies of first rust-resistance backcross generation; 10, 11, 12, and 13, progenies in the F_2 generation.

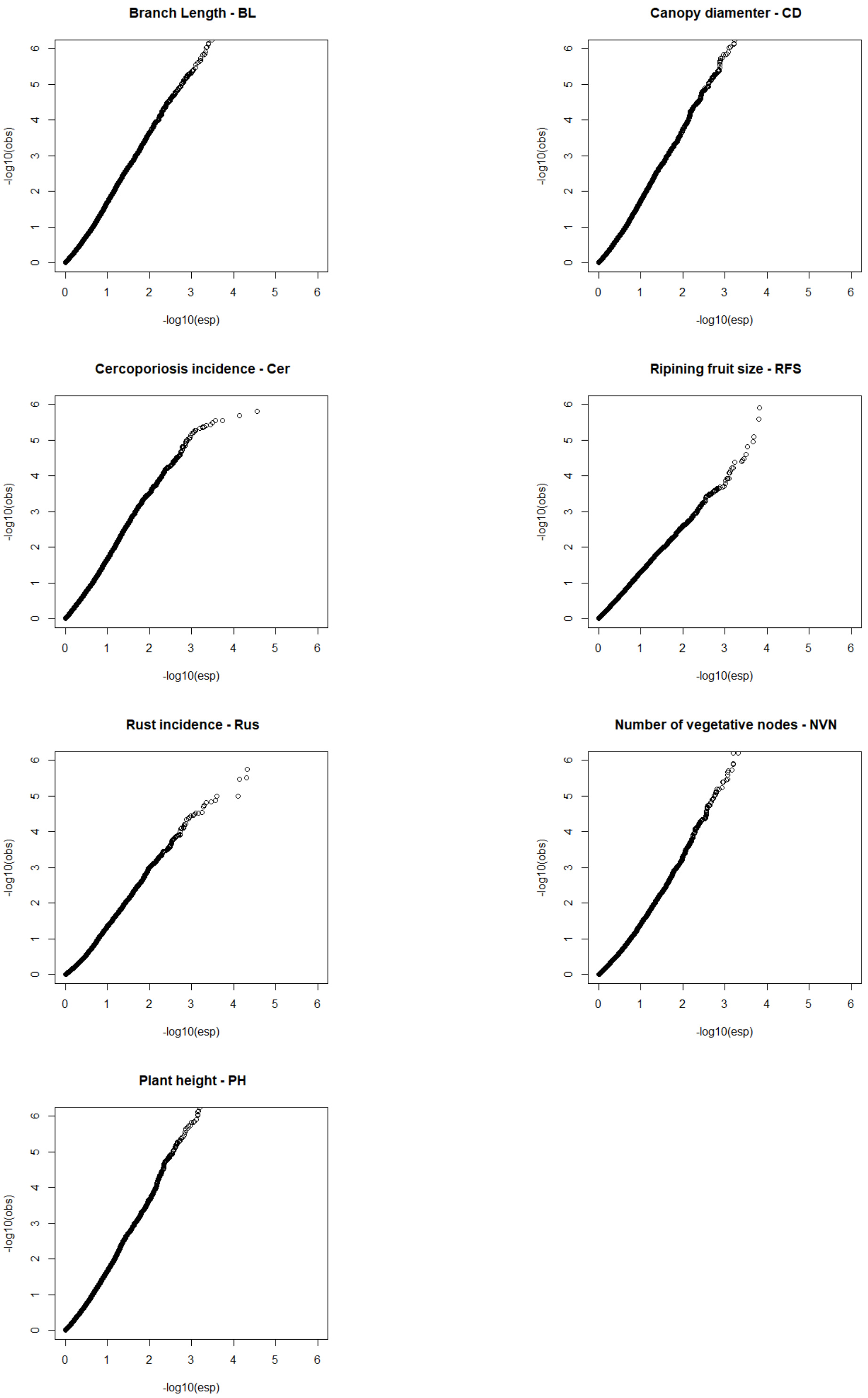

Figure S5. Q-Q plot for assessing GWAS results of traits with significant associations.

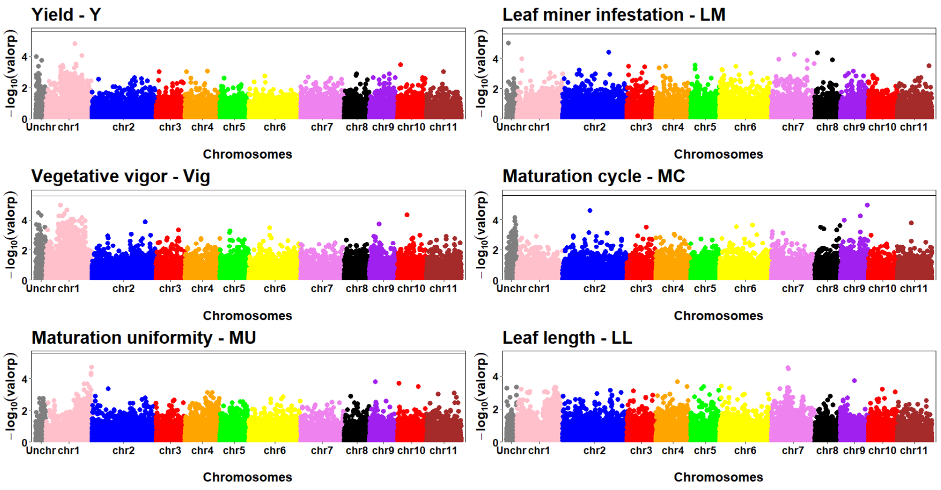

Figure S6. Manhattan plots for the traits. Y: yield; leaf miner infestation: LM; Vig: vegetative vigor; MC: maturation cycle; MU: maturation uniformity; LL: leaf length.

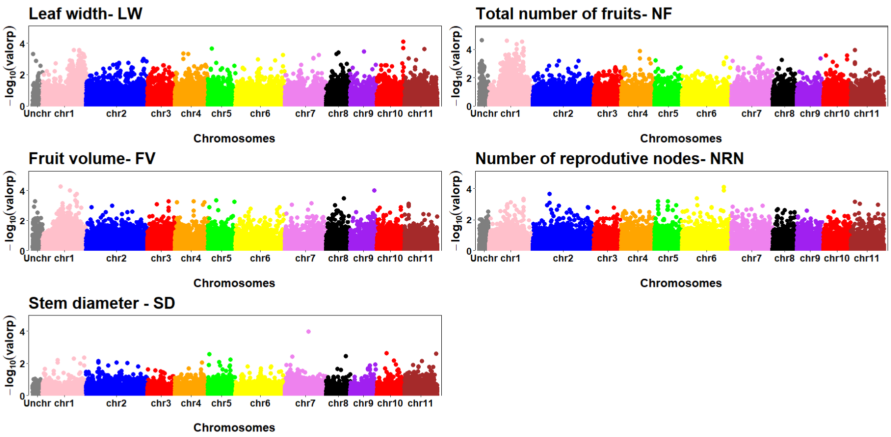

Figure S7. Manhattan plots for the traits. LW: leaf width; NF: total number of fruits; FV: fruit volume; NRN: number of reproductive nodes; SD: stem diameter.
